# A Human–Robot Interaction Perspective on Assistive and Rehabilitation Robotics

**DOI:** 10.3389/fnbot.2017.00024

**Published:** 2017-05-23

**Authors:** Philipp Beckerle, Gionata Salvietti, Ramazan Unal, Domenico Prattichizzo, Simone Rossi, Claudio Castellini, Sandra Hirche, Satoshi Endo, Heni Ben Amor, Matei Ciocarlie, Fulvio Mastrogiovanni, Brenna D. Argall, Matteo Bianchi

**Affiliations:** ^1^Institute for Mechatronic Systems, Mechanical Engineering, Technische Universität Darmstadt, Darmstadt, Germany; ^2^Human Centered Robotics Group, SIRSLab, Department of Information Engineering and Mathematics, University of Siena, Siena, Italy; ^3^Department of Mechanical Engineering, Abdullah Gul University, Kayseri, Turkey; ^4^Unit of Neurology and Clinical Neurophysiology, Department of Medicine, Surgery and Neuroscience, Section of Human Physiology, University of Siena, Siena, Italy; ^5^Institute of Robotics and Mechatronics, DLR German Aerospace Center, Oberpfaffenhofen, Germany; ^6^Technische Universität München, Munich, Germany; ^7^Interactive Robotics Laboratory, School of Computing, Informatics, and Decision Systems Engineering, Arizona State University, Tempe, AZ, United States; ^8^Department of Mechanical Engineering, Columbia University, New York, NY, United States; ^9^Department of Informatics, Bioengineering, Robotics and Systems Engineering, University of Genova, Genova, Italy; ^10^Department of Electrical Engineering and Computer Science, Northwestern University, Evanston, IL, United States; ^11^Department of Mechanical Engineering, Northwestern University, Evanston, IL, United States; ^12^Department of Physical Medicine and Rehabilitation, Northwestern University, Evanston, IL, United States; ^13^Rehabilitation Institute of Chicago, Chicago IL, United States; ^14^Research Centre “Enrico Piaggio”, University of Pisa, Pisa, Italy; ^15^Department of Information Engineering, University of Pisa, Pisa, Italy

**Keywords:** human–robot interaction, human-oriented design, learning and control, sensory feedback, affective computing, functional assessment, assistive and rehabilitation robotics

## Abstract

Assistive and rehabilitation devices are a promising and challenging field of recent robotics research. Motivated by societal needs such as aging populations, such devices can support motor functionality and subject training. The design, control, sensing, and assessment of the devices become more sophisticated due to a human in the loop. This paper gives a human–robot interaction perspective on current issues and opportunities in the field. On the topic of control and machine learning, approaches that support but do not distract subjects are reviewed. Options to provide sensory user feedback that are currently missing from robotic devices are outlined. Parallels between device acceptance and affective computing are made. Furthermore, requirements for functional assessment protocols that relate to real-world tasks are discussed. In all topic areas, the design of human-oriented frameworks and methods is dominated by challenges related to the close interaction between the human and robotic device. This paper discusses the aforementioned aspects in order to open up new perspectives for future robotic solutions.

## 1 Introduction

Recently, an increasing research interest in assistive and rehabilitation robotics is observed as a result of their greater capabilities in supporting and augmenting users. This stems from societal needs such as applications in healthcare, for example, mobility aids for people who are aging or with motor impairments (Dollar and Herr, 2008; Windrich et al., 2016), or in industry, such as the augmentation of workers carrying heavy loads (Dollar and Herr, 2008; Yan et al., 2015). Even though device designs have the functionality to perform desired tasks, many robotic devices demonstrate limited effectiveness not only due to technical limitations (Dollar and Herr, 2008; Yan et al., 2015; Windrich et al., 2016) but also due to insufficient knowledge about the human (Yan et al., 2015). Thus, assistive and rehabilitation robotics research and applications appear to require human-oriented approaches, since critically the devices interface with humans (Yan et al., 2015; Christ and Beckerle, 2016). Therefore, considering both technical and human aspects is crucial, and techniques from neural and human sciences should be considered beyond engineering techniques (Yan et al., 2015; Christ and Beckerle, 2016). Taking into account safety, functionality, effectiveness, and acceptance requires the collaboration of disciplines like mechatronics, computer science, biomechanics, neuroscience, and psychology. A focus on aspects of human–robot interaction (HRI) and interface technologies is needed, not the least in order to promote a pathway to systematic frameworks that consider, in both development and operation, the challenges of interfacing with the human.

In this perspective paper, the organizers and speakers of the *2nd Workshop on Human-Oriented Approaches for Assistive and Rehabilitation Robotics* (HUMORARR 2016) reflect and extend their discussions to point out directions for future research. The workshop comprised talks and discussion from experts working on assistive/rehabilitation robotics and/or human–robot interaction and was held in conjunction with the *IEEE International Symposium on Robot and Human Interactive Communication* (IEEE RO-MAN 2016) in New York, USA, on August 26, 2016.

This perspective covers four topics: control design, machine learning, sensory feedback, and affective computing, as well as reliability and assessment. Scientific issues determined in the workshop are formulated, and their relevance and potential are discussed.

## 2 Control Design (Matei Ciocarlie, Brenna D. Argall, Fulvio Mastrogiovanni, Claudio Castellini, Heni Ben Amor, and Philipp Beckerle)

One goal for assistive robotic devices is to not only “fit like a glove” but also to be just as easy to use as such a simple garment: the user simply dons the device, which starts doing its job without demanding additional cognitive load from the human. However, many existing assistive machines are burdensome to operate, or use input modalities that are easily accessible to non-impaired people but difficult for their target population.

Reducing (or maybe even eliminating) training time can speed up adoption and allow valuable therapist time to be used more effectively. One way to achieve this is to employ learning methods that incrementally adapt to the subject, situation, and environment (Castellini et al., 2015). Another possibility is to introduce shared autonomy to traditionally human-operated assistive machines (Argall, 2014, 2015; Jain et al., 2015) since semiautonomous operation can off-load cognitive and physical load from subjects to the machine and thereby increase user independence. Furthermore, the literature suggests the use of force and tactile information (Dahiya et al., 2010) for contact modeling and recognition as well as for motion analysis and planning, since these robotic devices will be in contact with the user (Cannata et al., 2010; Menguc et al., 2014). Therefore, perceptual and cognitive factors such as the classification of large-scale contacts (Muscari et al., 2013) or non-verbal communication signals should be taken into account.

A key challenge in semiautonomous solutions is how to appropriately share control between the robotic device and the human: the autonomous behavior should be predictable, providing assistance when appropriate and yet never taking control when undesired by the user (with the exception being cases of safety) (Argall, 2014; Erdogan and Argall, 2017; Gopinath et al., 2017). Furthermore, it is challenging how to adapt this control sharing over time, as the human’s abilities and preferences change.

The input provided by the human user presents significant challenges in itself: the human users and the robotic autonomy often do not provide control signals in the same space (Broad and Argall, 2016), and decoding user intent is a formidable task, especially if the output space is high dimensional. However, new advances in sensing (touch detectors, electromyographic sensors strain gages, etc.) and processing tools (dimensionality reduction, regression and classification, pattern recognition, etc.) are bringing us closer to that goal (Castellini et al., 2014).

An example of rapidly evolving technologies that could help in the design of appropriate human–robot interaction are tactile sensing and robot skins, which could acquire contact information from large-scale surfaces (Muscari et al., 2013; Denei et al., 2015; Youssefi et al., 2015a,b). Such robot skins need to be conformable, cheap, and easy to manufacture (Anghinolfi et al., 2013; Bianchi et al., 2016a; Le et al., 2016). Furthermore, tactile-based sensing and control cannot be seen as isolated features of novel rehabilitation devices. On the contrary, the way contact information is processed and the associated robot motion strategies must be informed by top-down rehabilitation or assistive policies. For example, given a contact regime, robot motions could be compliant in certain conditions, whereas they may be such to exert forces to implement a given rehabilitation tasks on other situations. Sensor skins meeting these requirements will enable robotic devices to physically interact with humans according to high-level policies and goals. Beyond obvious benefits that robot skin can provide to rehabilitation devices, it is noteworthy that they can also serve as an open benchmark to investigate cognition processes associated with non-verbal communication. These are related to the understanding of affective gestures such as constrained motions and are of the utmost importance in carrying out exercise routines in the most useful way.

## 3 Machine Learning (Claudio Castellini, Satoshi Endo, Sandra Hirche, Heni Ben Amor, and Philipp Beckerle)

Whenever confronted with a new tool, human beings learn to use it and adapt to it. Through neural plasticity and behavioral adaptation, exercise quantity and regularity can influence movement capabilities and alter the requirements for assistance or rehabilitation from a robotic device. Thus, robotic devices might guide users to carry out a desired exercise routine that is continually adapted to account for the individual user’s needs, in order to stimulate training and maximize its effect.

Recent literature in assistive robotics and prosthetics tries to explore and quantify this phenomenon. For instance, myoelectric signals are observed to distinctly change over days and weeks when used by subjects to control a hand prosthesis (Powell and Thakor, 2013). Novel muscle strategies are elicited by engaging people in a video game controlled *via* surface electromyography (Ison and Artemiadis, 2015). When such strategies are retained after weeks and months, this adaptation can be highly beneficial for control strategies derived *via* machine learning techniques, since signals become more separated and repeatable (Bunderson and Kuiken, 2012).

As human users and control algorithms adapt, exploiting potential co-adaptation to achieve mutual interaction is a promising research challenge. Besides observing and quantifying the phenomenon, considering it in algorithm design could promote a faster and more complete restoration of lost functions. One way of exploiting co-adaptation is to add to the training data synthetic patterns of activation based upon prior knowledge; even if such patterns do not match the current subject’s activations, the subject itself will adapt to them (Nowak and Castellini, 2016).

Mutual interaction is also a promising advancement for robot-assisted exercises: through physiotherapy training or progression of motor deficits, co-adapting interaction strategies that predict user behavior based on data-driven models are needed. Deep learning, for example, has recently gained much interest in robotics applications due to its expressive power and capability to predict overt human behavior (Hartford et al., 2016). Yet, other stochastic modeling techniques such as Gaussian processes can flexibly be designed to additionally address underlying sensory-motor functions important for neurorehabilitation such as neuromechanics of the human motion (Medina et al., 2016).

## 4 Sensory Feedback and Affective Computing (Matteo Bianchi, Claudio Castellini, and Philipp Beckerle)

For the effectiveness of robotics-enabled aides, sensory feedback can be as important as actuation, and especially haptic feedback due to the importance of touch in everyday life. In prosthetics, for instance, the natural action-perception loop is interrupted and the resulting lack of sensory information from the external environment is probably one of the main causes of device abandonment (Biddiss and Chau, 2007). However, trying to reproduce the richness of haptic information through artificial human–robot interfaces is a daunting task. Although studies exist on uninjured subjects which confirm that detailed multipoint feedback may help (Patel et al., 2016), the simultaneous delivery of too much information in general does not increase task execution performance. Especially whenever visual feedback is present, non-visual feedback is usually disregarded, and in extreme cases the simultaneous delivery of multiple types of information could even degrade performance (Kim and Colgate, 2012). Generally, appropriate feedback is the basis for successful multisensory integration that can facilitate embodiment of the device by the user (Giummarra et al., 2008; Christ et al., 2012a).

A possible strategy to tackle this issue could be the theoretical framework of sensory synergies (Bicchi et al., 2011). From the sensing point of view, we can identify an analogous mapping between low-level sensory variables (e.g., mechanoreceptors) to stabilize high-level human percepts, similar to motor synergies (Latash, 2008; Santello et al., 2016). Thereby, one might exploit that humans integrate data from multiple sensors to produce a coherent perceptual representation. Synergy approaches can be considered reductionist, since it aims at moving from the biological patterns underpinning human perception toward mathematical models. This aims at explaining how our brain produces low-dimensional perceptual representations from the abundance of sensors distributed in our body (Hayward, 2011). Therefore, computational approaches such as the tactile flow model that considers the flow of strain energy density can be applied (Bicchi et al., 2008). Understanding and mathematically modeling these sensory mechanisms could help to characterize mappings between cutaneous stimulation, motor acts, and proprioception (Moscatelli et al., 2016). Such a representation scheme could help to identify the most useful information for users for task accomplishment and to develop simple, effective, and intuitive interfaces for human–robot interaction (Bianchi and Serio, 2015). This is particularly important in assistive and rehabilitation robotics, where engineers need to know which information is most important for task accomplishment and how this relates to sensing and feedback.

Another important but scarcely considered aspect is the emotional response that assistive and rehabilitation robots can elicit in users, especially if feedback is provided. In the study by Bianchi et al. (2016c), a general framework to evaluate the emotional counterpart of haptic stimuli is developed. This framework is based on the circumplex model of affect (CMA) (Posner et al., 2005) and shows that both discriminative and affective haptic systems are able to elicit emotional responses that correlate with stimulus parameters.

Emotional quantification through CMA can also be associated with physiological measures related to the autonomous nervous system (ANS), which is directly connected to evoked human emotions (Bianchi et al., 2016b). Possible measures are electroencephalographic activity, heart rate variability, respiration dynamics, or electrodermal response (Schmidt et al., 2013; Valenza et al., 2016). The acquired data might feed new design approaches for robotic devices (Christ et al., 2012b; Beckerle, 2014) and, likely, increase user acceptance. Furthermore, considering ANS data in device control could enable estimating subtler but important aspects such as stress, fatigue, comfort, and motivation. Using such estimates, natural transfers between human and artificial control in shared control applications could be reached (Bianchi et al., 2016b; Gopinath et al., 2017). To calibrate such techniques and verify the effectiveness of the proposed methods, human-in-the-loop techniques are promising (Beckerle et al., 2012; Caspar et al., 2015) in regard to the embodiment of devices.

## 5 Reliability and Assessment (Gionata Salvietti, Domenico Prattichizzo, Simone Rossi, Claudio Castellini, and Philipp Beckerle)

Standards to evaluate the assistive and rehabilitative facets of novel robotic devices are still lacking. This is due mainly to the complexity of assessing user improvement that is attributable to the robot (Lo et al., 2010). Despite the availability of methods for assessing user improvements during rehabilitation (Gresham et al., 1997), standard protocols that go beyond usability questionnaires are missing for assistive devices: the gold standard for functional assessment would be a quantitative comparison of the gain reached in certain activities with and without the device. Such an assessment is, however, a complex task due to the sheer variety of assistive devices, as reviewed in the studies by Dollar and Herr (2008), Yan et al. (2015), and Windrich et al. (2016).

Toward the definition of standard assessment procedures, future approaches could adapt classical rehabilitation tests to examine assistive devices in the early stages of, or after, intense treatment. As an example, the Frenchay Arm Test (Heller et al., 1987) is used to quantify the number of successful actions with assistance (Salvietti et al., 2016). Alternatively, task or action sequences to be performed with the device could be specified. For instance, a set of bimanual tasks is used to investigate device performance in activities of daily living (Hussain et al., 2016). Regarding the lower limbs, aspects such as biomechanical functionality or metabolic cost seem to be promising (Au et al., 2009).

Although machine learning seems to be an effective method for control (Castellini et al., 2014), its reliability is still arguable (Jiang et al., 2012): it can work perfectly in the laboratory, but then largely fail in real-world settings. Regarding algorithmic reliability, incremental learning (Castellini, 2016) and shared autonomy might lead to a new generation of devices that interact with the human subject. Hence, future functional assessment protocols will need to take incrementality and interactivity into account (Castellini et al., 2015).

Finally, improving wearability would help to increase the use of devices in unstructured environments, and thereby support rehabilitation by extensive use in users’ homes. Passive and intrinsically compliant devices could foster wearability by combining safety, simplicity, and robustness with an excellent power-to-weight ratio (Wehner et al., 2013; Cappello et al., 2016). Furthermore, the device kinematics might differ from human ones; for example, this could relate to a robotic extra-finger that cooperates with the paretic arm of chronic stroke users to achieve a stable grasp and safe interaction in everyday tasks (Hussain et al., 2016; Salvietti et al., 2016).

## 6 Conclusion

Human–robot interaction plays an essential role in assistive and rehabilitation robotics. We have presented our perspective on the potential of, and also challenges within, this domain, from the standpoints of control, machine learning, sensory feedback, and affective computing, as well as reliability and assessment methods.

Regarding control, a key challenge is to design shared control or machine learning techniques that are predictable for users and do not override their demands. A grand topic in machine learning itself is mutual adaptation that allows users to explore and train the robotic aides themselves. Sensory feedback is a possibility to close human–machine control loops and could rely on models of basic sensory dimensions. Evaluation metrics going beyond questionnaires are scarce and functional assessment protocols that consider real-world task complexity and training progress are required, especially for learning devices. To tackle human–robot interaction systematically in design, human-oriented methods and human-in-the-loop experiments are promising topics for future research.

## Author Contributions

PB coordinated the writing process, the development of the paper structure, and integration of individual contributions. GS and RU supported this regarding the whole paper. All the authors contributed to the discussion in the perspective subsections, their names are mentioned in brackets after the subsection titles.

## Conflict of Interest Statement

The authors declare that the research was conducted in the absence of any commercial or financial relationships that could be construed as a potential conflict of interest.

## References

[B1] Anghinolfi D. Cannata G. Mastrogiovanni F. Nattero C. Paolucci M. (2013). On the problem of the automated design of large-scale robot skin. IEEE Trans. Autom. Sci. Eng. 10, 1087–1100. 10.1109/TASE.2013.2252617

[B2] Argall B. D. (2014). “Modular and adaptive wheelchair automation,” in Proceedings of the International Symposium on Experimental Robotics (ISER), Marrakech.

[B3] Argall B. D. (2015). “Turning assistive machines into assistive robots,” in Proceedings of the SPIE Photonics West Congress (San Francisco: Keynote).

[B4] Au S. K. Weber J. Herr H. (2009). Powered ankle-foot prosthesis improves walking metabolic economy. IEEE Trans. Robot. 25, 51–66. 10.1109/TRO.2008.2008747

[B5] Beckerle P. (2014). Human-Machine-Centered Design and Actuation of Lower Limb Prosthetic Systems. Aachen: Shaker Verlag.

[B6] Beckerle P. Christ O. Wojtusch J. Schuy J. Wolff K. Rinderknecht S. (2012). “Design and control of a robot for the assessment of psychological factors in prosthetic development,” in IEEE International Conference on Systems, Man and Cybernetics, Seoul.

[B7] Bianchi M. Haschke R. Büscher G. Ciotti S. Carbonaro N. Tognetti A. (2016a). A multi-modal sensing glove for human manual-interaction studies. Electronics 5, 42 10.3390/electronics5030042

[B8] Bianchi M. Valenza G. Greco A. Nardelli M. Battaglia E. Bicchi A. (2016b). “Towards a novel generation of haptic and robotic interfaces: integrating affective physiology in human-robot interaction,” in Robot and Human Interactive Communication (RO-MAN), 2016 25th IEEE International Symposium on (New York, NY: IEEE), 125–131.

[B9] Bianchi M. Valenza G. Lanata A. Greco A. Nardelli M. Bicchi A. (2016c). On the role of affective properties in hedonic and discriminant haptic systems. Int. J. Soc. Robot. 9, 87–95. 10.1007/s12369-016-0371-x

[B10] Bianchi M. Serio A. (2015). Design and characterization of a fabric-based softness display. IEEE Trans. Haptics 8, 152–163. 10.1109/TOH.2015.2404353 25720018

[B11] Bicchi A. Gabiccini M. Santello M. (2011). Modelling natural and artificial hands with synergies. Phil. Trans. R. Soc. B 366, 3153–3161. 10.1098/rstb.2011.0152 21969697 PMC3172595

[B12] Bicchi A. Scilingo E. P. Ricciardi E. Pietrini P. (2008). Tactile flow explains haptic counterparts of common visual illusions. Brain Res. Bull. 75, 737–741. 10.1016/j.brainresbull.2008.01.011 18394519

[B13] Biddiss E. Chau T. (2007). Upper-limb prosthetics: critical factors in device abandonment. Am. J. Phys. Med. Rehabil. Eng. 86, 977–987. 10.1097/PHM.0b013e3181587f6c 18090439

[B14] Broad A. Argall B. (2016). “Path planning under kinematic constraints for shared human-robot control,” in Proceedings of the International Conference on Automated Planning and Scheduling (ICAPS), London.

[B15] Bunderson N. E. Kuiken T. A. (2012). Quantification of feature space changes with experience during electromyogram pattern recognition control. IEEE Trans. Neural Syst. Rehabil. Eng. 20, 239–246. 10.1109/TNSRE.2011.2182525 22262686

[B16] Cannata C. Denei S. Mastrogiovanni F. (2010). “A framework for representing interaction tasks based on tactile data,” in IEEE International Symposium on Robot and Human Interactive Communication, Viareggio.

[B17] Cappello L. Binh D. K. Yen S.-C. Masia L. (2016). “Design and preliminary characterization of a soft wearable exoskeleton for upper limb,” in 6th IEEE International Conference on Biomedical Robotics and Biomechatronics (BioRob) (Singapore: IEEE), 623–630.

[B18] Caspar E. A. de Beir A. Magalhães Da Saldanha da Gama P. A. Yernaux F. Cleeremans A. Vanderborght B. (2015). New frontiers in the rubber hand experiment: when a robotic hand becomes ones own. Behav. Res. Methods 47, 744–755. 10.3758/s13428-014-0498-3 24942249

[B19] Castellini C. (2016). “Incremental learning of muscle synergies: from calibration to interaction,” in Human and Robot Hands, eds Bianchi M. Moscatelli A. (Switzerland: Springer International Publishing), 171–193.

[B20] Castellini C. Artemiadis P. Wininger M. Ajoudani A. Alimusaj M. Bicchi A. (2014). Proceedings of the first workshop on peripheral machine interfaces: going beyond traditional surface electromyography. Front. Neurorobot. 8:22. 10.3389/fnbot.2014.00022 25177292 PMC4133701

[B21] Castellini C. Bongers R. M. Nowak M. van der Sluis C. K. (2015). Upper-limb prosthetic myocontrol: two recommendations. Front. Neurosci. 9:496 10.3389/fnins.2015.00496 26778953 PMC4701912

[B22] Christ O. Beckerle P. (2016). Towards active lower limb prosthetic systems: design issues and solutions. Biomed. Eng. Online 15, 139 10.1186/s12938-016-0283-x 28105949 PMC5249020

[B23] Christ O. Beckerle P. Preller J. Jokisch M. Rinderknecht S. Wojtusch J. (2012a). The rubber hand illusion: maintaining factors and a new perspective in rehabilitation and biomedical engineering? Biomed. Eng. 57, 1098–1101. 10.1515/bmt-2012-4297

[B24] Christ O. Jokisch M. Preller J. Beckerle P. Rinderknecht S. Wojtusch J. (2012b). User-centered prosthetic development: comprehension of amputees’ needs. Biomed. Eng. 57, 1098–1101. 10.1515/bmt-2012-4306

[B25] Dahiya R. S. Metta G. Valle M. Sandini G. (2010). Tactile sensing – from humans to humanoids. IEEE Trans. Robot. 26, 1–20. 10.1109/TRO.2009.2033627

[B26] Denei S. Mastrogiovanni F. Cannata C. (2015). Towards the creation of tactile maps for robots and their use in robot contact motion control. Rob. Auton. Syst. 63, 293–308. 10.1016/j.robot.2014.09.011

[B27] Dollar A. M. Herr H. (2008). Lower extremity exoskeletons and active orthoses: challenges and state-of-the-art. IEEE Trans. Robot. 24, 144–158. 10.1109/TRO.2008.915453

[B28] Erdogan A. Argall B. D. (2017). The effect of robotic wheelchair control paradigm and interface on user performance, effort and preference: an experimental assessment. Robot. Auton. Syst. 10.1016/j.robot.2017.04.013

[B29] Giummarra M. J. Gibson S. J. Georgiou-Karistianis N. Bradshaw J. L. (2008). Mechanisms underlying embodiment, disembodiment and loss of embodiment. Neurosci. Biobehav. Rev. 32, 143–160. 10.1016/j.neubiorev.2007.07.001 17707508

[B30] Gopinath D. Jain S. Argall B. D. (2017). Human-in-the-loop optimization of shared autonomy in assistive robotics. IEEE Robot. Autom. Lett. 2, 247–254. 10.1109/LRA.2016.2593928 PMC6335047 30662953

[B31] Gresham G. E. Stason W. B. Duncan P. W. (1997). Post-Stroke Rehabilitation, Vol. 95 Collingdale, PA: DIANE Publishing.

[B32] Hartford J. S. Wright J. R. Leyton-Brown K. (2016). “Deep learning for predicting human strategic behavior,” in Advances in Neural Information Processing Systems, Vol. 29, eds Lee D. D. Sugiyama M. Luxburg U. V. Guyon I. Garnett R. (Barcelona: Curran Associates, Inc), 2424–2432.

[B33] Hayward V. (2011). Is there a ’plenhaptic’ function? Phil. Trans. R. Soc. B 366, 3115–3122. 10.1098/rstb.2011.0150 21969693 PMC3172594

[B34] Heller A. Wade D. Wood V. A. Sunderland A. Hewer R. L. Ward E. (1987). Arm function after stroke: measurement and recovery over the first three months. J. Neurol. Neurosurg. Psychiatry 50, 714–719. 10.1136/jnnp.50.6.714 3612152 PMC1032076

[B35] Hussain I. Salvietti G. Spagnoletti G. Prattichizzo D. (2016). The soft-sixthfinger: a wearable EMG controlled robotic extra-finger for grasp compensation in chronic stroke patients. IEEE Robot. Autom. Lett. 1, 1000–1006. 10.1109/LRA.2016.2530793

[B36] Ison M. Artemiadis P. (2015). Proportional myoelectric control of robots: muscle synergy development drives performance enhancement, retainment, and generalization. IEEE Trans. Rob. 31, 259–268. 10.1109/TRO.2015.2395731

[B37] Jain S. Farshchiansadegh A. Broad A. Abdollahi F. Mussa-Ivaldi F. (2015). “Assistive robotic manipulation through shared autonomy and a body-machine interface,” in Proceedings of the IEEE International Conference on Rehabilitation Robotics (ICORR), Singapore. 10.1109/ICORR.2015.7281253 PMC4737957 26855690

[B38] Jiang N. Dosen S. Müller K. R. Farina D. (2012). Myoelectric control of artificial limbs – is there a need to change focus? IEEE Sig. Process. Mag. 29, 152–150. 10.1109/MSP.2012.2203480

[B39] Kim K. Colgate J. (2012). Haptic feedback enhances grip force control of sEMG-controlled prosthetic hands in targeted reinnervation amputees. IEEE Trans. Neural. Syst. Rehabil. Eng. 20, 798–805. 10.1109/TNSRE.2012.2206080 22855230

[B40] Latash M. L. (2008). Synergy. Oxford: Oxford University Press.

[B41] Le T. H. L. Maiolino P. Mastrogiovanni F. Cannata C. (2016). Skinning a robot: design methodologies for large-scale robot skin. IEEE Robot. Autom. Mag. 23, 150–159. 10.1109/MRA.2016.2548800

[B42] Lo A. C. Guarino P. D. Richards L. G. Haselkorn J. K. Wittenberg G. F. Federman D. G. (2010). Robot-assisted therapy for long-term upper-limb impairment after stroke. N. Engl. J. Med. 362, 1772–1783. 10.1056/NEJMoa0911341 20400552 PMC5592692

[B43] Medina J. Endo S. Hirche S. (2016). “Impedance-based gaussian processes for predicting human behavior during physical interaction,” in Proceedings of the International Conference in Robotics and Automation (IEEE/ICRA), Stockholm.

[B44] Menguc Y. Park Y.-L. Pei H. Vogt D. Aubin P. M. Winchel E. (2014). Wearable soft sensing suit for human gait measurement. Int. J. Robot. Res. 33, 1748–1764. 10.1177/0278364914543793

[B45] Moscatelli A. Bianchi M. Serio A. Terekhov A. Hayward V. Ernst M. O. (2016). The change in fingertip contact area as a novel proprioceptive cue. Curr. Biol. 26, 1159–1163. 10.1016/j.cub.2016.02.052 27068417 PMC4865678

[B46] Muscari L. Seminara L. Mastrogiovanni F. Valle M. Capurro M. Cannata C. (2013). “Real-time reconstruction of contact shapes for large-area robot skin,” in IEEE International Conference on Robotics and Automation, Karlsruhe.

[B47] Nowak M. Castellini C. (2016). The LET procedure for prosthetic myocontrol: towards multi-DOF control using single-DOF activations. PLoS ONE 11:e0161678. 10.1371/journal.pone.0161678 27606674 PMC5015907

[B48] Patel G. Dosen S. Castellini C. Farina D. (2016). Multichannel electrotactile feedback for simultaneous and proportional myoelectric control. J. Neural. Eng. 13, 056015. 10.1088/1741-2560/13/5/056015 27618968

[B49] Posner J. Russell J. A. Peterson B. S. (2005). The circumplex model of affect: an integrative approach to affective neuroscience, cognitive development, and psychopathology. Dev. Psychopathol. 17, 715–734. 10.1017/S0954579405050340 16262989 PMC2367156

[B50] Powell M. A. Thakor N. V. (2013). A training strategy for learning pattern recognition control for myoelectric prostheses. J. Prosthet. Orthot. 25, 30–41. 10.1097/JPO.0b013e31827af7c1 23459166 PMC3581303

[B51] Salvietti G. Hussain I. Cioncoloni D. Taddei S. Rossi S. Prattichizzo D. (2016). Compensating hand function in chronic stroke patients through the robotic sixth finger. IEEE. Trans. Neural. Syst. Rehabil. Eng. 25, 142–150. 10.1109/TNSRE.2016.2529684 26890911

[B52] Santello M. Bianchi M. Gabiccini M. Ricciardi E. Salvietti G. Prattichizzo D. (2016). Hand synergies: integration of robotics and neuroscience for understanding the control of biological and artificial hands. Phys. Life Rev. 17, 1–23. 10.1016/j.plrev.2016.06.007 26923030 PMC5839666

[B53] Schmidt M. Penner D. Burkl A. Stojanovic R. Schümann T. Beckerle P. (2013). Implementation and evaluation of a low-cost and compact electrodermal activity measurement system. Measurement 92, 96–102. 10.1016/j.measurement.2016.06.007

[B54] Valenza G. Greco A. Citi L. Bianchi M. Barbieri R. Scilingo E. (2016). Inhomogeneous point-processes to instantaneously assess affective haptic perception through heartbeat dynamics information. Sci. Rep. 6, 28567. 10.1038/srep28567 27357966 PMC4928096

[B55] Wehner M. Quinlivan B. Aubin P. M. Martinez-Villalpando E. Baumann M. Stirling L. (2013). “A lightweight soft exosuit for gait assistance,” in Robotics and Automation (ICRA), 2013 IEEE International Conference on (Karlsruhe: IEEE), 3362–3369.

[B56] Windrich M. Grimmer M. Christ O. Rinderknecht S. Beckerle P. (2016). Active lower limb prosthetics: a systematic review of design issues and solutions. Biomed. Eng. Online 15, 5–19. 10.1186/s12938-016-0284-9 28105948 PMC5249019

[B57] Yan T. Cempini M. Oddo C. M. Vitiello N. (2015). Review of assistive strategies in powered lower-limb orthoses and exoskeletons. Rob. Auton. Syst. 64, 120–136. 10.1016/j.robot.2014.09.032

[B58] Youssefi S. Denei S. Mastrogiovanni F. Cannata C. (2015a). A real-time data acquisition and processing framework for large-scale robot skin. Rob. Auton. Syst. 68, 86–103. 10.1016/j.robot.2015.01.009

[B59] Youssefi S. Denei S. Mastrogiovanni F. Cannata C. (2015b). Skinware 2.0: a real-time middleware for robot skin. SoftwareX 3, 6–12. 10.1016/j.softx.2015.09.001

